# Solubility, pH-Solubility Profile, pH-Rate Profile, and Kinetic Stability of the Tyrosine Kinase Inhibitor, Alectinib

**DOI:** 10.3390/ph17060776

**Published:** 2024-06-13

**Authors:** Dheyaa Tohma Madlool, Israa Al-Ani, Tha’er Ata, Wael Abu Dayyih

**Affiliations:** 1 Faculty of Pharmacy, Al-Ahliyya Amman University, Amman 19328, Jordan; phdheyaatomeh@gmail.com (D.T.M.); thaerata7@gmail.com (T.A.); 2 Faculty of Pharmacy, Mutah University, Al-Karak 61710, Jordan; wabudayyih@mutah.edu.jo

**Keywords:** alectinib, non-small cell lung carcinoma, solubility, photodegradation, pH stability, pH solubility

## Abstract

Alectinib HCl (ALBHCl) is a tyrosine kinase inhibitor used for non-small cell lung carcinoma (NSCLC). The aim of this study is to unlock some of the physicochemical properties of ALBHCL that serve as a database for any future studies. A solubility study of ALBHCL was performed in different solvents. Also, photostability was tested in the solution and solid states, and the order of reaction and rate constant were calculated. In addition to the pH solubility relation, the pH-rate relation at different temperatures was also studied, and the profiles were constructed. A solubility study was also performed in different media for the purpose of optimizing suitable sink conditions for the in vitro dissolution testing of solid dosage forms. Solubility tests in multiple solvents and pH conditions revealed that the highest solubility was in DMSO, methanol, and chloroform, with acidic media yielding the maximum solubility but degrading at rather low pH levels. ALBHCL proved unstable at high temperatures and under light exposure, with varying stability across different pH levels. The optimal dissolution media for in vitro oral dosage form evaluation were determined, achieving sink conditions at pH levels of 6.8 and 4.5 with specific additives. This study enhances the existing database on ALBHCL’s physicochemical properties, emphasizing the importance of pH optimization in pharmaceutical processes and providing valuable insights into its pharmaceutical application.

## 1. Introduction

Lung cancer (LC), originating from the lung parenchyma or bronchi, stands as a leading cause of cancer-related deaths in the United States, surpassing even breast cancer in terms of female mortality rates [1]. According to the World Health Organization (WHO), the cancer burden continues to grow globally, exerting tremendous physical, emotional, and financial strain on individuals, families, communities, and health systems. Many health systems in low- and middle-income countries are least prepared to manage this burden, and large numbers of cancer patients globally do not have access to timely quality diagnosis and treatment [2].

Smoking is the primary cause of lung cancer, accounting for 90% of all cases, particularly in males. Other carcinogens like asbestos further elevate the risk. Passive smoking increases the risk of LC by 20–30% [3]. Additional risk factors include radiation exposure for other cancers, exposure to certain metals, and non-smoking-related causes like idiopathic pulmonary fibrosis. Furthermore, individuals with HIV demonstrate an increased LC risk, regardless of antiretroviral therapy or tobacco use [4].

Non-small cell lung cancer (NSCLC) encompasses various LC types, including large cell carcinoma, squamous cell carcinoma, and adenocarcinoma, with the latter being the most common [5].

Treatment of NSCLC: For stage 1 NSCLC, surgical resection, involving lobectomy or pneumonectomy with mediastinal lymph node biopsy, is the primary type of treatment, showing 78% and 53% 5 year survival rates for stages IA and IB, respectively [6]. Targeted therapy against specific mutations has emerged as a promising approach. Each mutation, such as epidermal growth factor receptors (EGFRs) and anaplastic lymphoma kinases (ALKs), has corresponding inhibitors like afatinib, gefitinib, and erlotinib for EGFR and ALBHCl HCl, crizotinib, and ceritinib for ALK, with crizotinib also approved for certain LC mutations [7].

Alectinib HCl (Alecensa^®^), chemically known as 9-ethyl-6,6-dimethyl-8-[4-(morpholin-4-yl)piperidin-1-yl]-11-oxo-6,11-dihydro-5H-benzo[b]carbazole-3-carbonitrile-hydrochloride, is a potent, selective ALK inhibitor used in treating ALK-positive advanced tumors, including lymphomas and recurrent or advanced NSCLC [8,9]. It inhibits anaplastic lymphoma kinase (ALK) and RET proteins by preventing their phosphorylation. Inhibition of ALK activation prevents downstream signaling of cell proliferation and decreases tumor survivability. ALBHCl has fivefold more potency for inhibiting ALK than crizotinib and maintains activity against many of the secondary mutants associated with resistance to crizotinib. ALBHCl has quite good blood-brain barrier penetration, with measured concentrations in the blood–cerebrospinal fluid (CSF) barrier approximately equal to the free concentration of ALBHCl in plasma [10].

Pharmacodynamic Properties: ALBHCl is a highly selective and orally active ALK inhibitor with an IC50 of 1.9 nM in cell-free assays and a dissociation constant of 2.4 nM in ATP-competitive assays for ALK [10]. It shows selective inhibition against few kinases, with significant effects on GAK, ALK, and LTK at 10 nM concentration [11]. Oral administration in tumor-bearing mice demonstrated dose-dependent tumor suppression, with an ED50 of 0.46 mg/kg and notable regression in the NCI-H2228 cell line. Its unique chemical structure enables it to overcome resistance to other ALK inhibitors, demonstrating efficacy against most crizotinib-resistant EML4-ALK mutations [12].

Pharmacokinetic Properties: ALBHCl dosages ranged from 20 to 300 mg every 12 h in a Japanese phase-one study, with 300 mg identified as the optimal dose. Peak plasma levels occurred 2.00–4.61 h after administration, with the AUC increasing linearly over 0–10 h [13]. CYP-3A4 primarily metabolizes ALBHCL, with most elimination occurring through feces [14]. Food intake minimally affects its pharmacokinetics, although non-fasting conditions increase the AUC and C_max_ by 20% [15].

The most common adverse drug reactions associated with ALBHCl are weight gain (9.9%), photosensitivity reaction (5.3%), stomatitis (3.3%), interstitial lung disease (1.3%), and drug-induced liver injury (1.3%). Also, nephrotoxicity was reported in rare cases [16]. Increased exposure to ALBHCl was also linked to bradycardia, as explained by Pruis et al. [17].

There is rather little information about the physicochemical properties of ALBHCL in the published literature. It has been mentioned that it is highly lipophilic [18] and soluble only in DMSO, without solubility studies on different solvents that might be used in research. Additionally, ALBHCL powder is delivered with the recommendation of protection from light without studies on its stability in light in its solution and solid states. These studies facilitate handling of this API on small and industrial scales. Such information is necessary to build a database for this API that makes future work easy and reliable.

The aim of this study is to provide pharmaceutically valuable information about ALBHCL as an API and its stability in light, different pH values, and temperature and specify the suitable dissolution media for the oral solid dosage form that achieves sink conditions, in addition to development and validation of an HPLC method for ALBHCL, which could be a good reference for future studies.

## 2. Results and Discussion

### 2.1. High-Performance Liquid Chromatography (HPLC) Analysis

**Figure 1:**
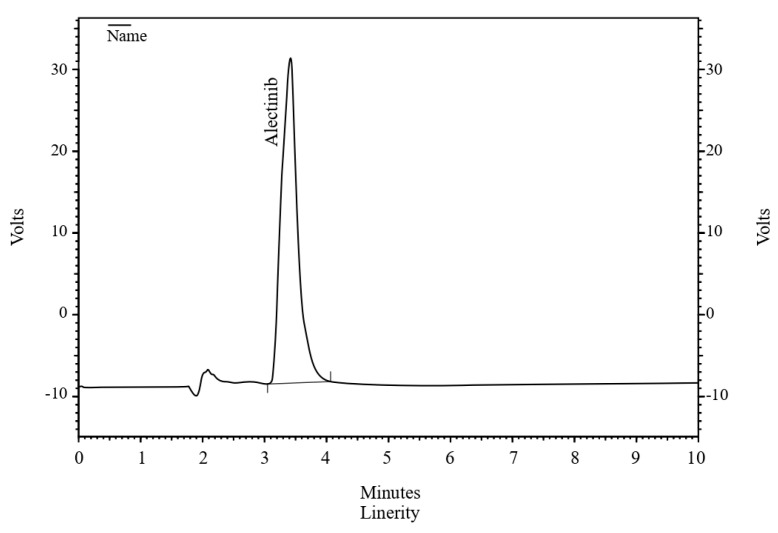
A chromatogram of ALBHCL showing a retention time of 3.5 min.

The chromatogram (Figure 1) illustrates the effective separation of ALBHCL, an aspect crucial for accurate quantification. This approach aligns with that of Lee et al. (2019) [8], who also used 339 nm for HPLC-PDA analysis of ALBHCL in human plasma. Interestingly, Prashanthi et al. (2018) chose 265 nm for ALBHCL analysis in bulk dosage forms, suggesting that optimal wavelengths may vary with the matrix and analytical conditions [19].

**Figure 2:**
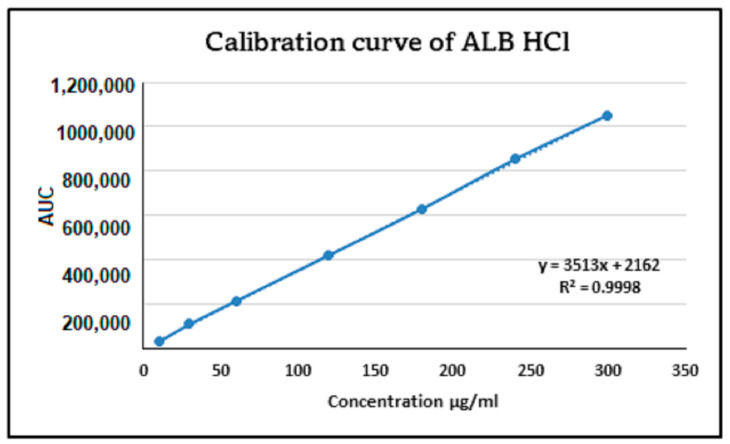
The linearity and calibration curve of ALBHCL, showing an R^2^ of 0.9998.

The linearity assessment of ALBHCL across a range of concentrations (from 10 µg/mL to 300 µg/mL) yielded an impressive correlation coefficient (R^2^) of 0.9998 (Figure 2). This high level of linearity is indicative of the method’s reliability for quantitative analysis. The RSD values, all below the 2% threshold set by ICH guidelines, further reinforce the method’s precision. Such high precision and accuracy are essential for ensuring the reliability of pharmaceutical analyses. The LOD was calculated to be 0.26 µg/mL, and the LOQ was calculated to be 0.79 µg/mL.

The precision and accuracy results (Table 1) demonstrate the method’s consistency over consecutive days, an important attribute for routine analytical applications. The accuracy range of 99.4–102.7% and RSD values within the acceptable limits, as per ICH guidelines, underscore the method’s suitability for pharmaceutical quality control.

The stability study (Table 1) underlines the importance of timely analysis of ALBHCL solutions, given their stability for only 24 h at room temperature. This finding has practical implications, particularly in ensuring the reliability of analytical results in routine laboratory practices.

### 2.2. Solubility Study of ALBHCL

**Figure 3:**
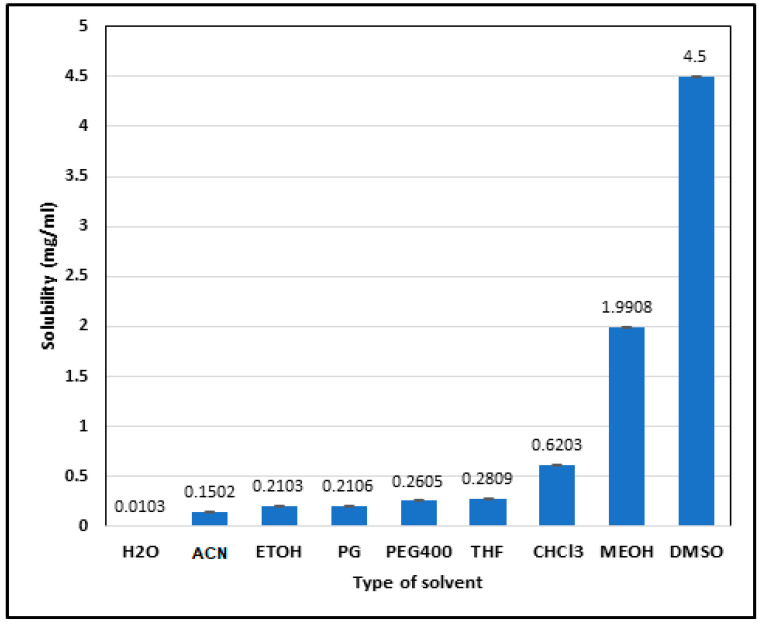
Solubility (in mg/mL) of ALBHCL in different solvents. (H_2_O = water; ACN = acetonitrile; ETOH = ethanol; PG = propylene glycol; PEG400 = polyethylene glycol; THF = tetrahydrofuran; CHCl_3_ = chloroform; MEOH = methanol; DMSO = dimethyl sulfoxide).

The solubility study (Figure 3) provided insights into ALBHCL’s solvent compatibility, which is crucial for formulating pharmaceutical preparations. The high solubility in DMSO and methanol aligns with its lipophilic nature (logP = 5.5), suggesting potential use in non-aqueous drug delivery systems. The absolute solubility in water was equal to 10.3 ± 1.2 µg/mL. Other solvents showed the following solubilities, all in µg/mL: methanol, 1990.8 ± 7.2; ethanol, 210.3 ± 4.5; acetonitrile, 150.2 ± 1.1; DMSO, 4500.0 ± 6.1; THF, 280.9 ± 2.4; chloroform, 620.3 ± 0.58; PEG400, 260.5 ± 6.0; and PG, 210.6 ± 5.8. The interaction between ALBHCL and the non-polar solvents as an induced dipole was due to the large non-polar area in the ALBHCL structure. However, the solubility of ALBHCL did not show a direct relation to the DEC of the solvent where DMSO, which has a DEC of 46, gave the highest solubility of ALBHCL among all options (*p* < 0.05). DMSO is known as a unique solvent with high solubilization power. The sulfur–oxygen link in DMSO has coordinated covalent single S+ → O− bonds, with both of the shared electrons coming from the sulfur. The molecular surface electrostatic potentials confirmed the highly negative characteristics of the oxygen and also revealed positive σ holes in the sulfur on the extensions of the O−S bonds [20].

The solubility of ALBHCl has been studied in different types of surfactants, such as transcutol, and different types of Capmul and Kolliphor as a part of the preparation of ALB SNEDDS by Park et al. The solubility of ALB HCL was found to be enhanced when using Kolliphor HS 15 and Capmul MCM C8 as the surfactant and oil, respectively, to formulate a stable dispersion [21].

### 2.3. pH Solubility Profile of ALBHCL

**Figure 4:**
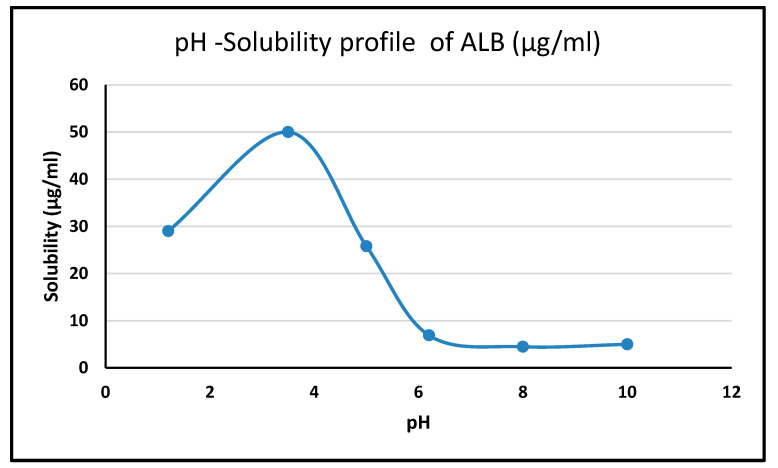
The pH solubility profile of ALBHCL, showing maximum solubility at pH 3.5.

**Figure 5:**
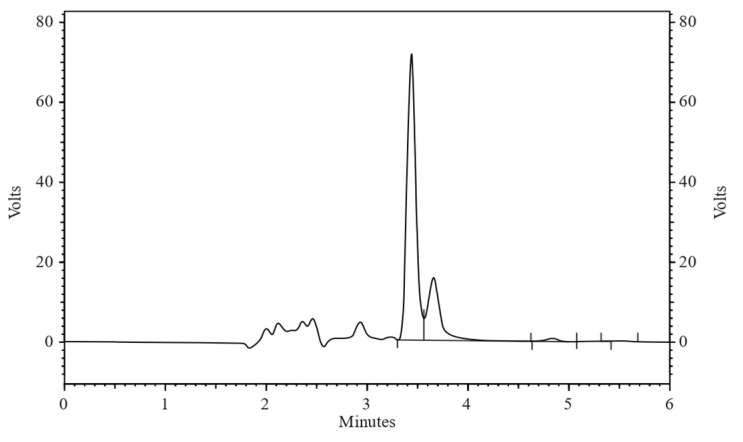
Chromatogram of ALBHCL at pH 1.2 after 24 h, showing a small peak adjacent to ALBHCL at an RT of 3.5 min.

The pH-dependent solubility (Figure 4) revealed ALBHCL’s instability at low pH levels, with its maximum solubility at pH 3.5. The high solubility in acidic media can be attributed to the protonation of the nitrogen atoms, making the molecular relatively more polar to associate with water molecules. At a pH of 1.2, ALBHCL showed degradation, which can be seen in the chromatogram (Figure 5) at the time of experimentation. This finding has implications for the formulation of ALBHCL, particularly in designing oral dosage forms where gastric stability is a concern.

At a pH of 3.5, ALBHCl showed the highest solubility of 50 µg/mL, and then at a pH of 5, it was 25.8 µg/mL. The solubility then decreased greatly at neutral and alkaline pH levels. The alectinib structure has four nitrogen atoms, and among them, the carbazole nitrogen is ionizable. In acidic pH levels, ionization increases, and solubility increases. With increased basicity, the nitrogen atom is unionized, and the solubility drops significantly. However, in all of these conditions, ALBHCl still has low solubility in water.

### 2.4. Photostability of ALBHCL

The photostability study demonstrated significant photodegradation of ALBHCL, necessitating light-protective measures during storage and handling. Only 32% of the intact ALBHCL remained after 36 h of exposure to light. This finding aligns with EMA’s guidance on Alecensa^®^ and emphasizes the importance of protecting photosensitive pharmaceuticals. Photodegradation is caused by the absorption of light, which might cause ionization or free radicle generation, which might result in initiation of a series of reactions of degradation. More evidence is required to identify the exact mechanism. The analysis of solid powder that was exposed to light showed a higher stability in light, where 82% remained intact after 36 h. The remaining log percent of ALBHCL was plotted against time and gave a straight line with a correlation of 0.95, which indicated that for the most part, the photodegradation followed a first-order pattern. Calculation of the rate constant of photodegradation as a first-order reaction gave K = 0.0829 h^−1^, and the half-life at room temperature of ALBHCL in a solution was 8.3 h. The solid powder photostability study showed stability for up to 36 h, with 99% recovery of ALBHCL from analysis of the powder.

### 2.5. Heat Stability of ALBHCL Solution

The heat stability study revealed a high activation energy for ALBHCL’s thermal degradation, suggesting its relative stability at lower temperatures. This information is crucial for storage and transport conditions to prevent thermal degradation.

Heat increases the kinetic energy of the molecules in the solution and facilitates the breakdown of liable bonds. Many materials, particularly organic materials, can undergo thermal degradation when exposed to high temperatures. This process can involve oxidative reactions with the environment or decomposition reactions that occur internally within the material.

**Figure 6:**
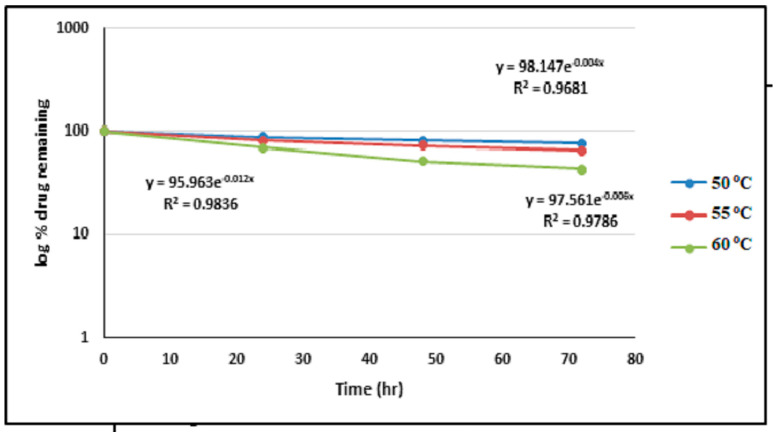
The log percentage remaining vs. a T plot of the photodegradation of ALBHCL in a solution, showing the first-order pattern of degradation.

Calculation of the rate constant of degradation as a first-order reaction (R^2^ = 0.69) gave the following rate constants: K50 °C = 0.0101332 h^−1^, K55 °C = 0.013818 h^−1^, and K60 °C= 0.0257936 h^−1^. These were plotted on an Arrhenius plot (Figure 6).

The Arrhenius equation relates the rate constant (K) of a reaction to the temperature (T) and the activation energy Ea [22]:

K = A e^(−Ea/RT)^

where K is the rate constant, A is the pre-exponential factor (frequency factor), Ea is the activation energy, R is the gas constant (8.314 J/(mol·K)), and T is the absolute temperature.

From the plot, the equation is y = −10.034 x +26.419, where y is lnK, x is 1/T, and the slope is −Ea/R:

−10.034 × 1000 = −Ea/8.3 J·M^−1^K^−1^

Ea = 83,000 J·M^−1^ Or 83 KJ/M

Alternatively, by using two K values at two temperatures, we have the following:

Ln K2/K1 = (−Ea/R)(1/T2^−1^/T1)

Ln (2.5459) = (−Ea/8.3) (−0.000096)

0.93 = Ea × 9.6 × 10^−5^/8.3

Ea = 80,000 J/mol or 80 KJ/M

This is the activation energy of degradation of ALBHCL by heat. Now, the rate constant at 25 °C can also be calculated with the same equation:

Ln (0.02579/K1) = (−83,000/8.3) (0.003003 − 0.0033557)

Ln (0.02579/K1) = 3.527

0.02579/K1 = 34.02

K1 (at 25 °C) = 7.5 × 10^−4^ h^−1^

Therefore, the shelf-life of an ALBHCL solution in methanol is 0.105/K_25_ = 140 h, and the half-life is 924 h.

In the context of thermal degradation, an activation energy of 80 kilocalories per mole (kJ/mol) is generally considered relatively high. The activation energy for a reaction gives an indication of the energy barrier that must be overcome for the reaction to occur. In the case of thermal degradation, a higher activation energy suggests that a significant amount of energy is required to initiate the degradation process.

This implies that the degradation of compound X is not likely to occur rapidly at lower temperatures, as the molecules need a substantial amount of thermal energy to reach the activation energy and proceed with the degradation. Reactions with high activation energies are often more temperature-sensitive and may require elevated temperatures to proceed at a noticeable rate [23].

### 2.6. pH Stability and pH-Rate Profile of ALBHCL

**Figure 7:**
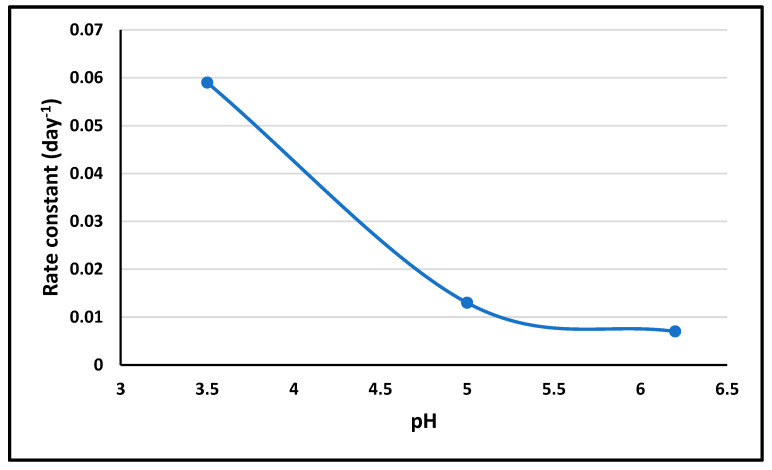
Partial pH-rate profile of ALBHCL using 3 pH values.

The pH stability study showed differential stability across pH values, informing the choice of the optimal pH levels for ALBHCL formulations based on the intended application and stability requirements. The results showed that increasing the pH would increase stability. However, the solubility study gave the opposite results, which make it necessary to choose an optimum pH level while dealing with ALBHCL in solution form. Figure 7 shows the pH-rate profile of ALBHCL.

At a pH of 1.2, ALBHCL showed degradation during testing, as shown by the chromatogram (Figure 5). This is why data of stability were not included in the profile.

### 2.7. Sink Conditions for Dissolution Testing of ALBHCL

The exploration of sink conditions is fundamental for accurate dissolution testing of ALBHCL. The identification of suitable media compositions achieving sink conditions is crucial for ensuring the reliability of dissolution studies, particularly for oral dosage forms. The results show that the dissolution media that fulfilled the criteria of 2–7 times the solubility of the amount in solid dosage form (150 mg) were phosphate buffer 6.8 + 2% SLS (M3), phosphate buffer 6.8 + 5% Tween 80 (M6), acetate buffer 4.5 + 2% SLS (M11), and acetate buffer 4.5 + 5% Tween 80 (M14). This means that one of these media could be suitable for dissolution testing with ensured sink conditions.

The research also identified that sink conditions for dissolution testing of ALBHCL in pH levels of 6.8 and 4.5 can be achieved by adding either 2% sodium lauryl sulphate (SLS) or 5% Tween 80. This is a crucial aspect for ensuring accurate and reliable dissolution testing of ALBHCL in pharmaceutical products.

## 3. Materials and Methods

### 3.1. Materials

This study utilized ALBHCL sourced from Biosynth, Coruna, Spain. Various reagents and chemicals, including methanol from Merck, ethanol from Labchem Laboratory Chemicals in the USA, acetonitrile from CarlouErba Reagents, Bernolsheim, France, and phosphoric acid from Carbon Group, Lincoln, UK, were employed. Additionally, a range of substances including sodium lauryl sulfate, potassium dihydrogen phosphate, disodium hydrogen phosphate, sodium hydroxide, and triethyleamine from Tedia, Ohio, USA and citric acid, sodium citrate, and Tween 80 from Loba Chemie, New Delhi, India were used.

### 3.2. Instruments

For precise measurements and analysis, we used a range of instruments: a balance from Phoenix Instrument (Garbsen, Germany), an oven from Memmert, (Schwabach, Germany), a pH meter from Lenway (Shenzhen, China) a water bath from Memmert (Schwabach, Germany), a hot plate and magnetic stirrer from Vision Scientific (Daejeon, South Korea), and an HPLC system from Finnigan Surveyor of Thermo Electron Corporation in Waltham, MA, USA. The HPLC system included components like a solvent delivery system pump (LC Pump Plus), a UV-VIS plus detector, and an autosampler plus. An Elmasonic S100 sonicater, a Shimadzu Irprestige 21-Spectrometer FTIR, a SHARP fridge (Osaka, Japan), and membrane filters from AXIVA were also utilized. A Hipersil column from Runcorn, UK (150 mm × 4.6 mm, 5 µm) was employed for chromatographic analysis.

### 3.3. Development and Validation of HPLC Method of Analysis for ALBHCL

Using the HPLC system mentioned above, the chromatographic conditions were as follows: a Hipersil C18- column (150 mm × 4.6 mm, 5 µm) and a mobile phase consisting of methanol:acetonitrile:orthophosphoric acid at a pH of 3.8 and a ratio of 40:30:30% *v*/*v*. The flow rate was 1 mL/min, and the column was maintained at ambient temperature. The injection volume was 20 µL, and the total run time was 7 min. The UV-VIS detector was set at a wavelength of 340 nm based on UV scanning of the compound and data reported in the literature [24]. The rinsing solution used was methanol:water (1:1). The method was validated in terms of precision (using 120 µg/mL), linearity (from 10 to 300 µg/mL), accuracy, limit of detection, and quantification. Also, short-term stability of the ALBHCL solution in methanol at a concentration of 50 µg/mL for 24 h was ensured on the bench at ambient temperatures.

### 3.4. Solubility Study in Water and Different Solvents

There is a lack of data regarding the solubility of ALBHCL in different solvents. Also, regarding solubility in water, it is known as “practically insoluble”. Knowing the exact solubility of ALBHCL would be of value. Two milliliters of each solvent (methanol, ethanol, acetonitrile, dimethyl sulfoxide (DMSO), tetrahydrofuran (THF), chloroform, polyethylene glycol 400 (PEG 400), and propylene glycol (PG)) were put in a glass test tube, and an excess of ALBHCL was put into each one and closed tightly. Then, they were covered with aluminum foil to exclude the light effect. Then, all tubes were put in a water bath at 25 ± 1 °C and 50 rpm for 48 h to reach equilibrium. After that, the samples were filtered using a 0.22 µm filter, and 1 mL was injected into the HPLC. Any sample with a high reading was diluted with the mobile phase.

### 3.5. pH-Solubility Profile

The effect of the pH level on the aqueous solubility of ALBHCL was studied by examining the solubility in different pH values (chloride buffer: pH 1.2; citrate buiffer: pH 3.5, 5, and 6.2; phosphate buffer: pH 8; and NaOH buffer: pH 10). All buffers were at a concentration of 0.05 M. The experiment was performed in the same way as the previous experiment on solubility.

### 3.6. Photostability

ALBHCL is known by manufacturing companies as “photosensitive”, and it is recommended to cover it with foil and use amber glass. But, there are no accurate data about the stability of the powder and solution. Here, 50 mL of ALBHCL solution in methanol was prepared by dissolving 2.5 mg of ALBHCL. The samples were put in a lab near natural sunlight for one month. The samples were withdrawn at different time intervals and measured by HPLC for the undegraded ALBHCL. The chromatogram was also examined. The same conditions were applied to 100 mg of ALBHCL in a small transparent glass container for solid state testing. Then, 2 mg was dissolved for each reading in methanol and measured by HPLC for the ALBHCL at different time intervals and concentrations through examination of the chromatogram.

### 3.7. Effect of Heat and Accelerated Stability Study of ALBHCL Solution

The stability of ALBHCL against heat and the rate of degradation was examined with an accelerating stability study. Solutions of ALBHCl in methanol were prepared and kept at 3 elevated temperatures: 50 °C, 55 °C, and 60 °C. The samples were measured at different time intervals to follow the concentrations of intact ALBHCL. Then, the rate constant of degradation at each temperature was calculated, and the shelf life of the solution at each temperature was calculated.

### 3.8. pH-Rate Profile

A citrate buffer at three pH values was prepared as described above (pH of 3.5, 5, and 6.2). Since ALBHCL has low solubility in water, a methanolic buffer was prepared by mixing 5 mL of the buffer with 5 mL of methanol, and 2.5 mg ALBHCL was added and dissolved. The initial concentration of ALBHCL was measured, and then readings were taken at different time intervals up to 2 weeks. The aim was to calculate the rate constant at each pH level and plot it against the pH values.

### 3.9. Long-Term Stability of ALBHCL Solution at Room Temperature

The solution was stored at room temperature for three months. (The temperature in the lab was fixed to 26 °C). The samples were measured at time zero, 2 weeks, and after 3 months, and the concentration of intact ALBHCL was recorded.

### 3.10. Study of Sink Conditions for the Dissolution Test at pH of 6.8 and 4.5

The sink conditions are necessary to perform a successful dissolution test for oral solid dosage forms. The sink conditions are approved when the solubility of API in the suggested dissolution media is 3–7 times the solubility of the whole dose in the media. Phosphate buffer (pH of 6.8) and acetate buffer (pH of 4.5) were prepared. Then, SLS, Tween 80, and ethanol were tried in different concentrations to achieve the target. Excess ALBHCL was added, and the samples were put in a water bath at 37 °C for 24 h (50 rpm) and analyzed. The suggested media were as shown in Table 2.

## 4. Conclusions

This study successfully established a reliable and validated high-performance liquid chromatography (HPLC) method with a UV detector for the analysis of ALBHCl. The solubility of ALBHCL was highest in dimethyl sulfoxide (DMSO), reaching 4500.0 ± 6.1 µg/mL, followed by methanol, with a solubility of 1990.8 ± 7.2 µg/mL. The study also highlighted that ALBHCL exhibits instability when exposed to light in solution form, with a short half-life of approximately 8.5 h. However, in its solid state, ALBHCL demonstrated greater stability under light exposure. Additionally, ALBHCL showed instability in highly acidic media (pH of 1.2) but had increased solubility in mildly acidic conditions (pH of 3.5 and 5) compared with more basic environments (pH of 8 and 10). This finding is critical for the storage, handling, and formulation of ALBHCL-based medications. Since ALBHCL is BCS class IV, attempts to enhance its solubility via different approaches like complexation and solid dispersion will be tried. Also, increasing the permeability through loading on mucoadhesive systems and micro- and nanoemulsions might be beneficial. All of these are parallel projects being executed by our team.

## Figures and Tables

**Table 1:** Inter- and intraday precision, accuracy, injection precision, and short-term stability results for ALBHCL.

Day 1 Measurements (120 µg/mL)			
Average area (n = 6)	Average conc. (µg/mL)	Accuracy (%)	RSD
426,564.33	122.05	101.7	0.6
Day 2 Measurements (120 µg/mL)			
423,890.5	121.2667	101.06	0.9
Injection Precision (120 µg/mL)			
Average area (n = 10)	Average conc. (µg/mL)	Accuracy (%)	RSD
419,831.8	120.12	100.1	0.62
24 h Stability of ALBHCL Solution in Methanol (50 µg/mL)			
Average area (n = 3) (24 h)	Average conc. (µg/mL)	Accuracy (%)	RSD
152,889	44.13	98.1	0.21

**Table 2:** Composition of the suggested media to prove sink conditions.

Code	Type and Content of Media	Code	Type and Content of Media
M1	Phosphate Buffer 6.8 + 0.5% SLS	M9	Acetate buffer 4.5 + 0.5% SLS
M2	Phosphate Buffer 6.8 + 1% SLS	M10	Acetate buffer 4.5 + 1% SLS
M3	Phosphate Buffer 6.8 + 2% SLS	M11	Acetate buffer 4.5 + 2% SLS
M4	Phosphate Buffer 6.8 + 0.5% Tween 80	M12	Acetate buffer 4.5 + 0.5% Tween 80
M5	Phosphate Buffer 6.8 + 1% Tween 80	M13	Acetate buffer 4.5 + 1% Tween 80
M6	Phosphate Buffer 6.8 + 5% Tween 80	M14	Acetate buffer 4.5 + 5% Tween 80
M7	Phosphate Buffer 6.8 + 5% Ethanol	M15	Acetate buffer 4.5 + 5% Ethanol
M8	Phosphate Buffer 6.8 + 10% Ethanol	M16	Acetate buffer 4.5 + 10% Ethanol

## Data Availability

All data of work is included in this paper.
